# Selecting Target Antigens for Cancer Vaccine Development

**DOI:** 10.3390/vaccines8040615

**Published:** 2020-10-17

**Authors:** Luigi Buonaguro, Maria Tagliamonte

**Affiliations:** Laboratory of Innovative Immunological Models, Istituto Nazionale per lo Studio e la Cura dei Tumori, “Fondazione Pascale”—IRCCS, 80131 Naples, Italy; l.buonaguro@istitutotumori.na.it

**Keywords:** tumor antigens, cancer vaccines, heteroclitic peptides

## Abstract

One of the principal goals of cancer immunotherapy is the development of efficient therapeutic cancer vaccines that are able to elicit an effector as well as memory T cell response specific to tumor antigens. In recent years, the attention has been focused on the personalization of cancer vaccines. However, the efficacy of therapeutic cancer vaccines is still disappointing despite the large number of vaccine strategies targeting different tumors that have been evaluated in recent years. While the preclinical data have frequently shown encouraging results, clinical trials have not provided satisfactory data to date. The main reason for such failures is the complexity of identifying specific target tumor antigens that should be unique or overexpressed only by the tumor cells compared to normal cells. Most of the tumor antigens included in cancer vaccines are non-mutated overexpressed self-antigens, eliciting mainly T cells with low-affinity T cell receptors (TCR) unable to mediate an effective anti-tumor response. In this review, the target tumor antigens employed in recent years in the development of therapeutic cancer vaccine strategies are described, along with potential new classes of tumor antigens such as the human endogenous retroviral elements (HERVs), unconventional antigens, and/or heteroclitic peptides.

## 1. Introduction

Preventive vaccines were originally developed to elicit an antigen-specific memory immunity in healthy subjects that is able to promptly react to subsequent infections by pathogens that could occur during the lifetime. Conversely, cancer vaccines are a therapeutic strategy aimed at eliciting a specific de novo immune response against tumor antigens or amplifying an existing anti-tumor immune response. Therefore, in addition to a memory immunity, such vaccines are intended to elicit a potent anti-cancer effector immune response. This mechanism involves the professional antigen-presenting cells (APCs) for triggering a cytotoxic effector CD8^+^ T-cell (CTL) response.

Several therapeutic cancer vaccine strategies and formulations have been evaluated in recent years in different tumor settings involving thousands of cancer patients. However, only modest effects have been reported at a low rate (less than 7%) and an overall rate of clinical benefit of around 20% [1,2,3,4]. The only FDA-approved therapeutic cancer vaccine to date is Provenge^®^ for patients with castration-resistant prostate cancer, which showed a limited 4.5-month improvement in overall survival (OS) compared to the placebo [5,6].

Such limited efficacy may be ascribed to two main factors: the immunosuppressive factors infiltrating the tumor microenvironment (TME) and the specificity of target tumor antigens included in the vaccine formulation. While the first factor can be addressed by designing strategies combining cancer vaccines and other immunotherapies [7,8,9,10,11], the latter requires the identification of novel tumor-specific antigens able to elicit effective and specific anti-tumor responses [12,13]. Tumor antigens need to be sufficiently distinct from self-antigens to break the immunological tolerance that physiologically blocks undesired auto-immune reactivity against normal cells.

In the present review, target tumor antigens employed in recent years in the development of therapeutic cancer vaccine strategies are described, together with potential new classes of tumor antigens, such as the human endogenous retroviral elements (HERVs), as unconventional antigens and/or heteroclitic peptides.

## 2. Tumor Antigens

Cancer vaccines are based on tumor antigens expressed in the context of Major Histocompatibility Complex (MHC) molecules able to elicit a strong tumor-specific CTL response, which may result in the killing of tumor cells and cancer regression.

Early cancer vaccines were based on approaches aimed at targeting the broadest antigen repertoire to avoid selection of escape variants. These included autologous tumor lysates, whole tumor-derived mRNA, irradiated autologous tumor cells, or allogeneic tumor cell lines, and have been evaluated in several clinical trials targeting different tumor types [14,15]. However, these approaches have several limitations, such as the need for a sufficient amount of tumor specimen, their collection and formulation, and challenges in terms of logistics and standardization of regulatory demands, including Good Manufacturing Practice (GMP) requirements. One of the most problematic aspects is related to the overwhelming number of non-tumor self-antigens in the whole tumor cell preparation, which not only dilutes the number of tumor-specific antigens but also induces an immunological tolerance [16]. To overcome such limitations, in recent years, therapeutic cancer vaccines are mostly based on one or a restricted number of cancer antigens. Tumor antigens can be classified into tumor-associated antigens (TAAs) and tumor-specific antigens (TSAs). In particular, TSAs are considered more effective than TAAs in cancer immunotherapy because they are the only cancer-specific targets unique to cancer cells, deriving either from viral antigens or from tumor-specific genomic mutations [17].

### 2.1. Tumor-Associated Antigens (TAAs)

Cancer cells, as result of their malignant profile, can constitutively overexpress antigens derived from protein, which are mainly involved in the replication and/or migration of the cancer cells. The antigens derived from the aberrantly overexpressed self-antigens in tumor cells compared to normal cells (e.g., RAGE-1, hTERT, HER2, mesothelin, and MUC-1) are defined as tumor-associated antigens (TAAs) and might represent universal antigens among patients with the same malignancy [18,19,20,21]. Besides the overexpressed antigens, TAAs can include: cell lineage differentiation antigens, which are normally not expressed in adult tissue (e.g., tyrosinase, gp100, MART-1, prostate-specific antigen (PSA); prostatic acid phosphatase (PAP)) [22,23,24]; and cancer/germline antigens (also known as cancer/testis), which are normally expressed only in immune privileged germline cells (e.g., MAGE-A1, MAGE-A3, NY-ESO-1, and PRAME) [25,26,27,28,29].

Overexpressed and tissue differentiation antigens are able to induce an antitumor immune response when high levels of expression of these proteins reach the threshold for T cell recognition, breaking immunological tolerance. However, the main drawback with using TAAs in cancer immunotherapy is the potential induction of autoimmunity against the corresponding normal tissues [30,31]. As these antigens are also expressed in healthy tissue as self-antigens, they are generally characterized by low immunogenicity, and T cells have low affinity receptors (TCR), which are unable to mediate effective anti-tumor responses [32]. Additionally, T cells that recognize these antigens may be removed from the immune repertoire by central and peripheral tolerance [33]. The formulation with an effective adjuvant may overcome the problem, significantly increasing the immunogenicity of the antigens and resulting in a clinical benefit for cancer patients.

Cancer germline/cancer testis antigens (CTAs) are tumor-associated antigens expressed only in human tumors of different histological origins but not in somatic normal tissue, except for testis and placenta tissue [34]. In this respect, CTAs have been considered promising targets for immunotherapy approaches thanks to their tumor-specificity and strong immunogenicity for the absence of immune tolerance.

#### TAA-Based Clinical Trials

Several therapeutic cancer vaccines based on TAAs have been evaluated in different phases of clinical trials addressing diverse malignancies. However, the induction of a strong CTL response has shown poor correlation with a favorable clinical outcome in several malignancies [35,36]. This may be due to different reasons, including the low affinity between the TCR and the antigens, the tumor evasion with loss of tumor antigen expression, and the inhibition of the cytotoxic activity in the immunosuppressive tumor environment.

Several clinical trials have been and are currently being conducted to assess the safety and immunogenicity of therapeutic cancer vaccines based on TAAs in different cancer settings [13]. In this respect, our group has coordinated a Phase I/II clinical trial assessing the safety and immunogenicity of a novel therapeutic cancer vaccine approach for Hepatocellular carcinoma (HCC) based on naturally processed and presented wild-type tumor-associated antigens (TAAs) (HepaVac-101 clinical trial, EudraCT Nr. 2015-003389-10) [37,38]. Of all these therapeutic cancer vaccine early-stage clinical trials, only five have been moved forward to Phase III efficacy trials and have been completed. Of these, only three have enrolled a sufficient number of patients to generate significant publishable data with limited efficacy (Table 1).

The trial evaluating the efficacy of Nelipepimut-S(NP-S) antigen in preventing breast cancer recurrence showed no serious adverse events (SAEs) and no significant between-arms differences in disease-free survival (DFS) events at the median follow-up (16.8 months). In the NP-S arm, however, imaging detected 54.1% of recurrence events in asymptomatic patients versus 29.2% in the placebo arm (*p* = 0.069), contributing to early trial termination [39].

The trial comparing the gp100 vaccine alone vs. the combination with Ipilimumab in patients with metastatic melanoma showed that the vaccine did not improve the overall survival as compared to Ipilimumab alone [40].

Finally, a clinical trial evaluated the efficacy of the gp100 combined with high-dose interleukin-2 (IL-2) in patients with metastatic melanoma. Although the experimental arm treated only with the gp100 vaccine was missing, the results showed that the vaccine provided an improved overall clinical response as well as longer progression-free survival compared to the immune-activating agent IL-2 alone [41].

### 2.2. Tumor-Specific Antigens (TSAs)

The limited results obtained with cancer vaccines based on TAAs urged the development of new strategies, in particular, the identification of different types of target antigens. Tumor-specific antigens (TSAs) are strictly specific to tumors not expressed on the surface of normal cells and include mutated neoantigens as well as antigens from oncoviruses, endogenous retroviral elements (HERVs), and unconventional antigens [17,42,43].

Mutated neoantigens are personalized antigens arising from cancer-related nonsynonymous mutations or other genetic alterations resulting in mutated peptides presented by HLA on the tumor cell surface of the immune system. Cancers are characterized by accumulation of genetic and epigenetic alterations in somatic cells with selective growth advantage to cancer cells. Driver mutations are therefore positively selected during the evolution of the cancer and cannot be lost because they are often required for maintenance of the final cancer. A direct consequence is the possible mutation of protein sequences and presentation of mutated antigens in the human leucocyte antigen-1 (HLA-I) complex on the tumor surface, different from the germline. Consequently, a specific and effective T cell response against cancer cells is triggered and not subject to central and peripheral immune tolerance [44]. The relevance of the mutated neoantigens in eliciting a potent anti-tumor T cell response is supported by several studies showing that response to the immune checkpoint inhibitors (ICI) often correlates with high tumor mutation load, which leads to a high number of mutated neoantigens [45].

RNA-sequencing (RNA-seq) data from The Cancer Genome Atlas (TCGA) from thousands of tumor samples show that the number of neoantigens per tumor type correlates positively with a gene expression signature of T cell cytolytic activity [43]. In particular, analysis of whole-exome sequencing of 619 colorectal cancers showed that high neoantigen load is associated with increased numbers of TILs and improved survival [46].

However, contradictory results have been recently reported about the correlation of the neoantigen load with the patients’ survival in other tumors [47,48,49,50]. In a recent study, we showed that neither the Tumor Mutational Burden (TMB) nor the number or the quality of the predicted neoantigens are associated with a prolonged survival in HCC patients not undergoing immunotherapy treatment. This contrasts results in melanoma and lung cancer patients undergoing immunotherapy [51,52]. These contradictory results might be due to the small number of mutations and low neoepitope load in HCC patients [53].

The major limitation of cancer vaccines based on mutated neoantigens is that they are strictly individual (private), and their identification requires a combination of high-throughput genomics, proteomics, and immunomics screening procedures, which currently cannot be applied on a large scale. Moreover, the efficacy of such a highly-personalized approach is possibly reduced by the high mutational rate of tumors, which drives a constant generation of new target mutated neoantigens and a consequent cancer immune evasion.

#### TSAs-Based Clinical Trials

Several clinical trials based on mutated neoantigens are currently ongoing in Phase I or Phase I/II, targeting a variety of cancer types (Table 2). Results have been published only from three clinical trials, and they all show the feasibility and safety of the strategy. In particular, melanoma patients treated with patient-specific mutated neoantigens responded to vaccination, discriminating between wild-type and mutated antigens. Vaccinated patients showed significantly delayed tumor recurrence and experienced complete tumor regression after anti-programmed cell death-1 (anti-PD-1) therapy, with the expansion of the repertoire of neoantigen-specific T cells [54,55,56]. The same approach applied to patients affected by glioblastoma showed safety and immunogenicity [57].

Feasibility and immunogenicity were confirmed in other recently completed clinical trials targeting different solid tumors [58], small cell of lung carcinoma (SCLC) [59], as well as melanoma [60].

### 2.3. Human Endogenous Retroviral Elements (HERVs) as Target Antigens

Human endogenous retroviral elements (HERVs) constitute 8% of the human genome and derive from the chromosomal integration of retroviral RNAs upon germline infections [61].

In cancer, transcription of HERVs is induced and activated upon malignant transformation and/or epigenetic therapy, such as DNA methyltransferase inhibitors (DNMTi) and histone deacetylase inhibitors (HDACi), becoming potential targets for cancer therapeutic approaches [62,63,64]. The combination of epigenetic therapies may be very effective in eliciting a strong and robust expression of a wide range of Endogenous retroviral elements (ERVs) and may induce the expression of ERV LTRs located within genes that then act as novel promoters generating novel transcripts [65].

Activation of the HERVs can lead to a state of viral mimicry, inducing an innate immune response and leading to production of type I and type III interferon and other cytokines [66,67]. By mimicking viral infections, ERVs could function as an intrinsic adjuvant, possibly sensitizing cancer cells for immune recognition [68]. One of the consequences of activated interferon signaling is the transcriptional induction of antigen presentation machinery, including the major histocompatibility complex (MHC) class I alleles and the transporter involved in antigen processing 1 (TAP1) [69,70].

All these events lead to the generation of a novel pool of tumor-specific antigens identified in different tumor types that can be exploited as T cell targets on tumor cells [62]. HERV-derived antigens have been used to develop cancer vaccines and chimeric antigen receptor (CAR)-expressing T cells, which have been tested only in a pre-clinical setting to date [71,72,73,74,75,76]. Altogether, these pre-clinical studies show the safety, immunogenicity, and preliminary efficacy data, but none of these strategies have been tested in human clinical trials so far.

### 2.4. Unconventional Antigens

The identification of tumor antigens relies on the proteome sequencing by high-throughput LC−MS/MS analysis [77]. However, proteomics data from MS/MS spectra are interpreted using reference protein sequence databases and cannot be used to identify any novel cancer-specific sequences [78]. Therefore, only a strategy combining proteomics and genomics data from the same tumor lesion (i.e., proteogenomic) can enable the identification of tumor-specific peptides that are missing from the reference databases [79,80].

Identification of mutated neoantigens can significantly benefit from direct detection using proteogenomics [81]. Somatic mutations as well as gene fusions have been identified in colorectal [82], breast [83,84], ovarian [85], and liver cancers [86]. However, such a combined approach shows major potency in the identification of unconventional antigens, which otherwise would be difficult to prove. The proteasomes may generate peptide splice variants, splicing two peptide fragments together, significantly increasing the number of Human leukocyte antigen (HLA) ligands [87,88].

Strikingly, a single spliced peptide can arise from non-consecutive sequences even across multiple genes, leading to a great diversity of displayed HLA ligands, whose contribution to cancer immunology yet remains elusive [89,90].

Proteogenomics enables the identification of the proteome deriving from non-coding or unannotated RNAs. Several long non-coding RNAs can be translated by ribosomes into short proteic sequences, providing a potential source of HLA ligands [91,92]

Cancer-specific MHC class I antigens derived from the non-coding region have been described and proven to elicit anti-cancer CTL responses in mouse cancer models [93]. Unlike mutated neoantigens that arise from passenger mutations, non-coding RNA antigens are detected across individuals and may serve as attractive targets of vaccination or adoptive T cell transfer therapy [94].

## 3. Optimizing Antigenic Targets

To improve the immunogenicity of tumor antigens, mainly the TAAs, to be included in cancer vaccine formulations, peptides can be modified to increase their affinity and binding to the present MHC-I [95]. Such modified peptides (heteroclitic peptides) have been shown to break the immunological tolerance, inducing a more potent CD8^+^ T cell response able to recognize the native peptide expressed on the tumor cells and kill them [96,97,98,99,100]. The low affinity between the T cell receptor (TCR) and the peptide-major histocompatibility complex (pMHC) would allow the TCR to cross-react with multiple pMHCs [101,102,103].

### 3.1. Heteroclitic Peptides Improving Binding to MHC-I

Most of the studies have described an improvement of the CD8^+^ T cell response modifying the amino acid residues in the anchor positions interacting with the HLA molecule [99,100,104,105].

A peptide derived from gp100, a lineage differentiation antigen identified in melanoma, has been modified (heteroclitic) to optimize its bind to MHC complex. This modified peptide, gp100:209–217(210 M), binds with a higher affinity to HLA-A2 than the corresponding wt peptide and stimulates a specific and better T cell response in vitro and in vivo [97]. Clinical trials based on vaccination with 210 M antigen, alone or in combination with interleukin-2 (IL-2), have demonstrated the induction of peptide- and tumor-specific cytotoxic T-lymphocyte responses in peripheral blood [106,107]. In particular, a randomized phase III clinical trial, based on 210 M peptide vaccine, showed that in the group treated with gp100 peptide vaccine followed by high-dose interleukin-2, the response rate was higher and progression-free survival longer than in group treated with interleukin-2 alone [41].

Another modified peptide, CAP1-6D, an epitope of CEA, has been modified to improve the binding to MHC-I complex and has been shown to trigger a more potent CTL response, and T cells activated have been shown to be cross-reactive with wild-type CAP1 and to recognize CEA+ HLA-A2+ tumor cells [108,109].

### 3.2. Heteroclitic Peptides Improving Binding to TCR

An alternative approach for improving the immunogenicity of natural TAAs is to generate heteroclitic peptides with mutations in the TCR-binding residues to break the immunological tolerance and induce a more potent CD8^+^ T cell response [110,111]. Heteroclitic peptides modified in the TCR-binding residues of melanoma specific Trp2 TAA have been shown to improve the control of tumor growth [112]. Preliminary results from our group showed that recognition of a wild-type (WT) epitope by Peripheral blood mononuclear cells (PBMCs) can be significantly improved by modifying the TCR-facing amino acids, in particular at the P4 residue, of the HPV E7 WT epitope expressed on TC1 mouse lung tumor cell lines. Bioinformatics prediction algorithms identified specific amino acid substitutions at the P3 and P4 residues of the epitope, resulting in an increased affinity of the WT peptide to the H-2-Db allele. Moreover, heteroclitic peptides with amino acid changes in one of the TCR-facing and anchor position residues elicit an even stronger immune response, cross-reacting with the parental wild-type peptide. CTL elicited by the heteroclitic peptides show potent lytic activity on target cells expressing the WT peptide as well as control of tumor growth in vivo (in press).

## 4. Conclusions

Cancer immunotherapy has experienced tremendous progress in the last decade, including the dramatic expansion of our understanding of how cancer cells evade the immune system, and the development of several new therapies that are benefitting cancer patients.

Therapeutic cancer vaccines offer an attractive alternative immunotherapy because of their potential safety, specificity, and long-lasting response due to stimulation of immune memory.

**Figure 1:**
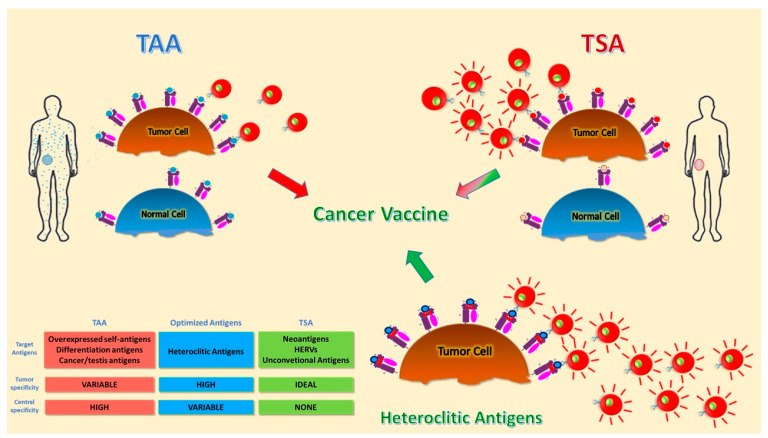
Schematic representation of different tumor antigens. Targets for tumor vaccines fall into tumor-associated antigens (TAAs) and tumor-specific antigens (TSAs). TAAs are self-antigens that are either preferentially or abnormally expressed in tumor cells, but may be expressed at some level in normal cells as well. T cells that bind with high affinity to TAAs are low in number because they are deleted by central and peripheral tolerance mechanisms. Heteroclitic peptides are modified TAAs able to break tolerance and induce a more potent T cell response. TSAs include antigens encoded only by cancer cells and are truly tumor-specific, eliciting high-affinity T cells. Different colors indicate the difference between antigens presented by normal and tumor cells. The red-lines on T cells indicate more activated and tumor-specific cells.

Unfortunately, many previous attempts to develop effective therapeutic cancer vaccines yielded disappointing results. Tumor antigens used so far all suffer from major drawbacks. TAAs suffer from expression on normal cells and immunological tolerance, which can be overcome by designing appropriate heteroclitic epitopes. TSAs represent the optimal target antigens but suffer from patient specificity, which hampers exploitation on a large scale. Unconventional antigens may represent a great advancement, and their efficacy needs to be proven in clinical trials (Figure 1). The accurate evaluation of the previous failures, combined with the constant technological improvements, will lead to the identification of the optimal tumor antigens. At the same time, the development of appropriate delivery strategies, adjuvants, and combination therapies to counteract the immunosuppressive tumor microenvironment will ultimately provide the sought improvement in the clinical outcome of cancer patients.

## Figures and Tables

**Table 1:** Cancer vaccines in Phase III completed based on tumor-associated antigens (TAAs).

Tumor	Peptide Vaccine	Status	Phases	NCT Number	Ref.
Breast Cancer	Nelipepimut-S (NP-S)	Completed	Phase 3	NCT01479244	[39]
Metastatic Melanoma	MDX-1379 (gp100)	Completed	Phase 3	NCT00094653	[40]
	gp100	Completed	Phase 3	NCT00019682	[41]
Multiple Myeloma	MAGE-A3/NY-ESO-1	Completed	Phase 2/Phase 3	NCT00090493	N.A.
Esophageal Cancer/Gastric Cancer	G17DT	Completed	Phase 3	NCT00020787	N.A.

**Table 2:** Cancer vaccines in Phase I/II based on tumor-specific antigens (TSAs).

Tumor	Status	Phases	NCT Number
ALL	Active, not recruiting	Phase 1|Phase 2	NCT03559413
Breast Cancer	Recruiting	Phase 1	NCT04105582
Fibrolamellar HCC	Recruiting	Phase 1	NCT04248569
Follicular Lymphoma	Not yet recruiting	Phase 1	NCT03361852
Gastric Cancers	Recruiting	Not Applicable	NCT03468244
Glioblastoma	Recruiting	Phase 1	NCT04015700
	Active, not recruiting	Phase 1	NCT03422094
	Recruiting	Phase 1	NCT02287428
	Completed	Phase 1	NCT02149225
HCC	Recruiting	Phase 1|Phase 2	NCT04251117
	Recruiting	Phase 1	NCT03674073
Kidney Cancer	Recruiting	Phase 1	NCT02950766
Lymphocytic Leukemia	Not yet recruiting	Phase 1	NCT03219450
Melanoma	Not yet recruiting	Phase 1|Phase 2	NCT04364230
	Recruiting	Phase 1	NCT04072900
	Not yet recruiting	Phase 1	NCT03929029
	Active, not recruiting	Phase 2	NCT02129075
	Completed	Phase 1	NCT02035956
	Active, not recruiting	Phase 1	NCT01970358
Multiple Cancers	Recruiting	Phase 1	NCT04147078
	Recruiting	Phase 1	NCT03956056
	Recruiting	Phase 1|Phase 2	NCT03953235
	Not yet recruiting	Not Applicable	NCT03908671
	Active, not recruiting	Phase 1	NCT03662815
	Recruiting	Phase 1|Phase 2	NCT03639714
	Recruiting	Phase 1	NCT03568058
	Recruiting	Phase 1	NCT04087252
	Recruiting	Phase 1	NCT03552718
NSCLC	Recruiting	Phase 1	NCT04078269
	Not yet recruiting	Phase 1	NCT03871205
	Recruiting	Phase 1	NCT04487093
	Recruiting	Phase 1	NCT04397926
Ovarian Cancer	Not yet recruiting	Phase 1	NCT04024878
Pancreatic Cancer	Recruiting	Phase 1	NCT04161755
	Recruiting	Phase 1	NCT03645148
	Active, not recruiting	Phase 1	NCT03122106
	Recruiting	Phase 1	NCT03558945
Pediatric Brain Tumor	Not yet recruiting	Phase 1	NCT03988283
	Not yet recruiting	Phase 1	NCT03068832
Prostate Cancer	Recruiting	Phase 1	NCT03532217
SCLC/NSCLC	Not yet recruiting	Phase 2	NCT04397003
	Not yet recruiting	Phase 1	NCT04266730
SPCM	Recruiting	Early Phase 1	NCT03631043
TNBC	Recruiting	Phase 1	NCT03199040
UBC	Recruiting	Phase 1	NCT03359239

ALL, acute lymphoblastic leukemia; HCC, hepatocellular carcinoma; NSCLC, non-small cell lung cancer; SCLC, small cell lung cancer; SPCM, smoldering plasma cell myeloma; TNBC, triple-negative breast carcinoma; UBC, urothelial/bladder cancer.
